# Colorimetric and Fluorescence Dual-Mode Biosensors Based on Peroxidase-Like Activity of the Co_3_O_4_ Nanosheets

**DOI:** 10.3389/fchem.2022.871013

**Published:** 2022-04-08

**Authors:** Jingying Tan, Weifu Geng, Junde Li, Zhen Wang, Shaohao Zhu, Xiuzhong Wang

**Affiliations:** ^1^ College of Chemistry and Pharmaceutical Sciences, Qingdao Agricultural University, Qingdao, China; ^2^ College of Plant Health and Medicine, Qingdao Agricultural University, Qingdao, China; ^3^ Hospital of Qingdao Agricultural University, Qingdao Agricultural University, Qingdao, China

**Keywords:** biosensor, dual modes, mimic enzyme, hydrogen peroxide, glucose

## Abstract

The mimic enzyme has become a research hotspot in recent years because of its advantages of high stability, convenient preparation, and low price. In this article, Co_3_O_4_ nanosheets synthesized by a simple hydrothermal method possess the characteristics of a peroxidase-like activity. The results demonstrated that 3,3′,5,5′-Tetramethylbenzidine (TMB) could be oxidized by H_2_O_2_ to produce a typical blue product (oxTMB) which has a strong absorption at 650 nm wavelength with the help of the Co_3_O_4_ nanosheets. Thus, a simple and sensitive colorimetric detection method for H_2_O_2_ was established with a good linear relationship (2–200 μM) and a low limit of detection (0.4 μM). Meanwhile, the colorimetric product can effectively quench the fluorescence emitted by Ru(bpy)_3_^2+^. Therefore, a colorimetric and fluorescence dual detection mode photochemical sensor for H_2_O_2_ detection is constructed based on the principle of the inner filter effect (IFE) between the colorimetric product (oxTMB) and Ru(bpy)_3_^2+^. It can effectively avoid the false positive problem of a single detection mode. In the presence of glucose oxidase, glucose can be catalyzed to produce gluconic acid and H_2_O_2_; therefore, the sensor can also be used for the determination of glucose with a good linear relationship (0.02–2 μM) and a low limit of detection (5 nM). Experimental results showed that the sensor has a high sensitivity and strong anti-interference ability which can be used for the detection of actual samples.

## Introduction

Enzymes are generally biological catalysts (or biocatalysts) that can accelerate the biochemical reactions in living organisms (Hemalatha et al., 2013; Meghwanshi et al., 2020). However, natural enzymes are usually made up of proteins (or RNAs); therefore these biocatalysts with high costs are vulnerable to inactivation (Sharma et al., 2021). They are often not optimal for practical applications (Lancaster et al., 2018). Thus, artificial enzymes have been developed by scientists as an alternative to natural enzymes (Li et al., 2014; Neelam et al., 2019). Since it was reported first that the Fe_3_O_4_ magnetite nanoparticles possess an intrinsic mimetic enzyme activity (Gao et al., 2007), nanozymes and nanomaterials with enzyme-mimicking activities have become a research hotspot in recent years due to their facile synthesis, tunable catalytic activities, high stability, and low cost (Lin Y. et al., 2014; Wang H. et al., 2019; Liang and Han, 2020). Many nanomaterials, such as cobaltosic oxide nanoparticles (Wang P. et al., 2019), manganese dioxide (Zou et al., 2021), graphene oxide hybrid (Wang Q. et al., 2021; Zhang et al., 2021e), nanoceria (Zhang J. et al., 2021), carbon dots (Li et al., 2022), VS_2_ (Huang et al., 2018), PtS_2_ (Zhang W. et al., 2021), MoS_2_ (Zhang X. et al., 2021; Tan et al., 2021), and WS_2_ (Nandu et al., 2021), had been shown to possess a similar peroxidase-like activity. These nanozymes had been used in various fields including biosensing (Wang L. et al., 2021; Liu et al., 2021), bioimaging (Gao et al., 2017; Liu et al., 2019), therapeutics (Chen et al., 2021; Zhang et al., 2021d; Zhang et al., 2021e; Hai et al., 2021), and biofuel cells (Le and Il, 2021) as substitutes for natural enzymes. Among these nanozymes, Co_3_O_4_ nanomaterials exhibit multienzyme activities at different pH conditions (Wang P. et al., 2019) which had been used to construct enzyme-free glucose sensors and other biosensing applications. Although various Co_3_O_4_ nanostructures such as nanoflowers, polyhedral, and spherical shapes have been successfully synthesized (Balouch et al., 2015; Liu et al., 2020; Cao et al., 2022), some disadvantages and several challenges in the synthetic routes need to be overcome, such as the need for special instruments, the cost and assisted agents, and the complicated process of the operation.

Two-dimensional (2D) layered nanomaterials have attracted an increasing research interest recently because they possess a larger surface area and more accessible active sites with a smaller diffusion barrier for the substrate molecules (Zhang et al., 2020; Hasan et al., 2021). There are few reports about 2D layered Co_3_O_4_ nanosheets in biosensing fields (Zhao et al., 2021). Herein, Co_3_O_4_ nanosheets were synthesized by a simple hydrothermal process, and the intrinsic peroxidase-like catalytic activity of the Co_3_O_4_ nanosheets has been discussed.

**Scheme 1:**
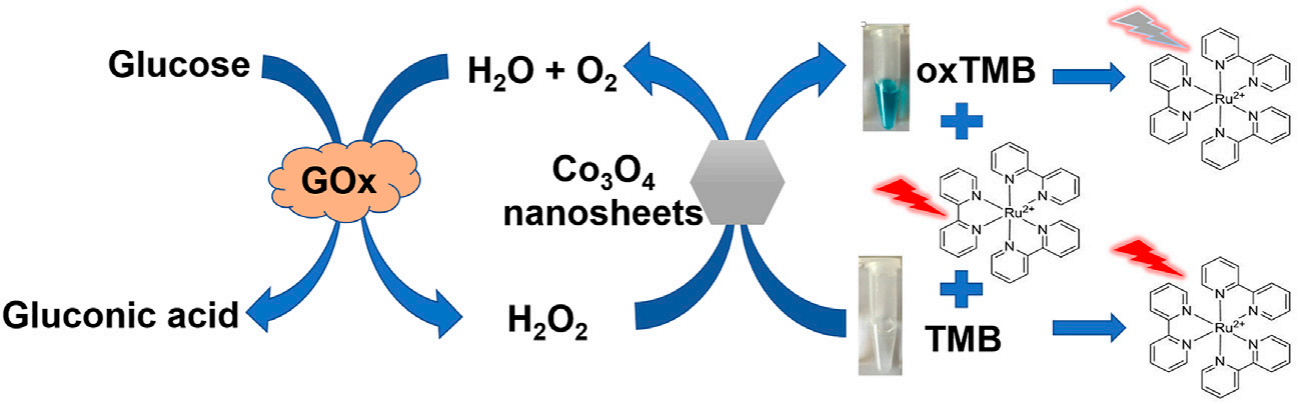
Schematic illustration for the determination of H_2_O_2_ and glucose based on Co_3_O_4_ nanozymes colorimetric/fluorescent dual mode sensing strategy.

As one of the reactive oxygen species (ROS), hydrogen peroxide (H_2_O_2_) plays critical roles in some biological processes, such as biosynthesis and cell signaling (Liu et al., 2015). However, abnormally elevated ROS levels can destroy redox homeostasis and cause oxidative stress and serious damage to the structure and function of macromolecules in the cell (Pratsinis et al., 2017; Lin et al., 2020). So excessive H_2_O_2_ is associated with the occurrence and development of many diseases. Several techniques have been used for the detection of H_2_O_2_. Among these techniques, colorimetric detection of hydrogen peroxide has been widely reported due to its obvious advantage of simplicity, visualization, and low cost. In this system, Co_3_O_4_ nanosheets can catalyze the oxidation of 3,3′,5,5′-Tetramethylbenzidine (TMB) to afford a blue oxidized TMB (oxTMB) form in the presence of H_2_O_2_. Thus, a simple and sensitive colorimetric method to detect H_2_O_2_ was established. Interestingly, the colorimetric product (oxTMB) with a strong absorption at 650 nm wavelength can effectively quench the fluorescence emitted by Ru(bpy)_3_^2+^. The inner filter effect (IFE) occurred between oxTMB and Ru (bpy)_3_^2+^ because the fluorescence emission spectrum of Ru (bpy)_3_^2+^ is from 550 to 750 nm (with a maximum emission wavelength at 610 nm) which is overlapped with the absorption spectrum of oxTMB. Therefore, a colorimetric and fluorescence dual-mode photochemical sensor for the detection of H_2_O_2_ is constructed based on the principle of the IFE. Glucose can be catalyzed to produce gluconic acid and H_2_O_2_; therefore, we further designed a sensitive and facile fluorescence sensor based on the Co_3_O_4_ nanosheets for the determination of glucose, which is one of the most common analysts providing an assessment of metabolic disorders and diabetes mellitus (Chaianantakul et al., 2018). It has been successfully applied for the determination of glucose in fruit juice and human blood samples (Scheme 1).

## Materials and Methods

### Chemicals and Materials

3,3′,5,5′-Tetramethylbenzidine (TMB), Co (NO_3_)_2_.6H_2_O, glucose, fructose, maltose, sucrose, ascorbic acid, and dopamine were purchased from Aladdin (Shanghai, China). Ruthenium bipyridine was purchased from Sigma-Aldrich (Shanghai, China). Hexamethylenetetramine, Na_2_HPO_4_, NaH_2_PO_4_, H_2_O_2_, acetic acid, sodium acetate, hydrochloric acid, and NaOH were purchased from Sinopharm Chemical Reagent Co., Ltd (Shanghai, China). All the chemical reagents were of analytical grade. Deionized water (18.2 MΩ cm at room temperature), obtained from a Milli-Q water purification system (Millipore Corp., MA), was used to prepared all of the aqueous solutions.

### Measurement and Apparatus

FT-IR spectra were obtained from the KBr slice with a Nicolet iS10 FT-IR spectrophotometer (Thermo Fisher Scientific, Shanghai). X-ray diffraction (XRD) patterns were recorded on a Bruker D8 Advance diffractometer (Germany) with a Cu Kα (1.5406 Å) radiation source. All pH measurements were performed with a Sartorius PB-10 digital pH meter (Shanghai, China). Transmission electron microscopy (TEM) and scanning electron microscopy (SEM) were performed with a HITACHI model HT7700 instrument operating at 80 kV accelerating voltage and a ZEISS Gemini 300 with OXFORD Xplore, respectively. The ultraviolet-visible (UV-Vis) absorption spectra were recorded by using a U3900 spectrophotometer (Hitachi, Japan). All fluorescence measurements were performed on a Hitachi F-7000 fluorescence spectrometer. The excitation wavelength was set at 496 nm, and the emission spectra from 550 to 750 nm were observed. The fluorescence intensity at 610 nm was used to evaluate the performance of the proposed strategy.

### Synthesis of Co_3_O_4_ Nanosheets

Co_3_O_4_ nanosheets were prepared in accordance with the method previously reported with a minor modification (Hwang et al., 2011). Under continuous stirring conditions, 1.3 g of Co(NO_3_)_2_·6H_2_O and 0.6 g of hexamethylenetetramine were dissolved in 100 ml of deionized water, respectively. Then, the aforementioned two solutions were mixed together. The resultant solution was maintained at pH 10 with 1 M sodium hydroxide solution and was vigorously stirred for 2 h. Next, the resultant solution was transferred into a pressure-tight teflon-lined stainless-steel autoclave, and then, it was heated up to 110°C. After 15 h, the autoclave cooled down naturally to room temperature. Then, the obtained products were washed with water, ethanol, and acetone, in turn, and vacuum dried at 60°C for 4 h. At last, the dried products were calcined for 2 h at 200°C. The obtained products were studied for their morphological characteristics and the peroxidase mimetic activity test.

### Kinetic Study and Peroxidase Mimetic Activity of Co_3_O_4_ Nanosheets

The Co_3_O_4_ nanosheets were added into HAc-NaAc buffer (100 mM, pH 4.5) in the presence of H_2_O_2_ by varying concentrations of TMB. The reactions were monitored in time at a wavelength 650 nm by using a U3900 spectrophotometer. The steady-state kinetic catalytic parameters were determined based on the Michaelis–Menten equation(Huang et al., 2018).

### Detection of H_2_O_2_ and Glucose Using the Co_3_O_4_/TMB/Ru(bpy)_3_^2+^ System

H_2_O_2_ detection was conducted as follows: different concentrations of H_2_O_2_ were introduced into the mixture of 20 μg/ml Co_3_O_4_ nanosheets and 0.5 mM TMB in HAc-NaAc buffer (100 mM, pH 4.5). The solution was incubated for 30 min at 37°C, and the absorbance of the solution was detected.

Glucose detection was conducted as follows: 5 μL of 50 mgmL^−1^ glucose oxidase was added into 95 μL PBS (10 mM, pH 7.4) containing different concentrations of glucose, and then, the mixture was incubated for 30 min at 37°C. Subsequently, the abovementioned reaction solution was transferred into 400 μL acetate buffer (100 mM, pH 4.5) containing 0.5 mM TMB, 20 μg/ml Co_3_O_4_ nanosheets, and 0.5 mM Ru (bpy)_3_^2+^. After incubating for another 30 min at 37°C, the reaction solution was monitored by using the fluorescence spectrophotometer. In the control experiments, fructose, maltose, sucrose, ascorbic acid, and dopamine were used instead of glucose, respectively.

For glucose determination in human serum samples, they were pretreated according to the literature (Peng and Weng, 2017) with a minor modification: 50 μL of the serum sample was diluted with 50 μL water and then were added into 900 μL solution containing 0.11 M Ba(OH)_2_ and 0.0765 M ZnSO_4_. The resultant solution was centrifuged for 10 min with 4,000 rpm. The supernatant was collected for the determination of glucose. All the experiments involving human beings were approved by the Ethics Committee Approval of China and operated in strict compliance with the Ethics Committee of Qingdao Agricultural University.

## Results and DISCUSSION

### Synthesis and Characterization of Co_3_O_4_ Nanosheets

**FIGURE 1:**
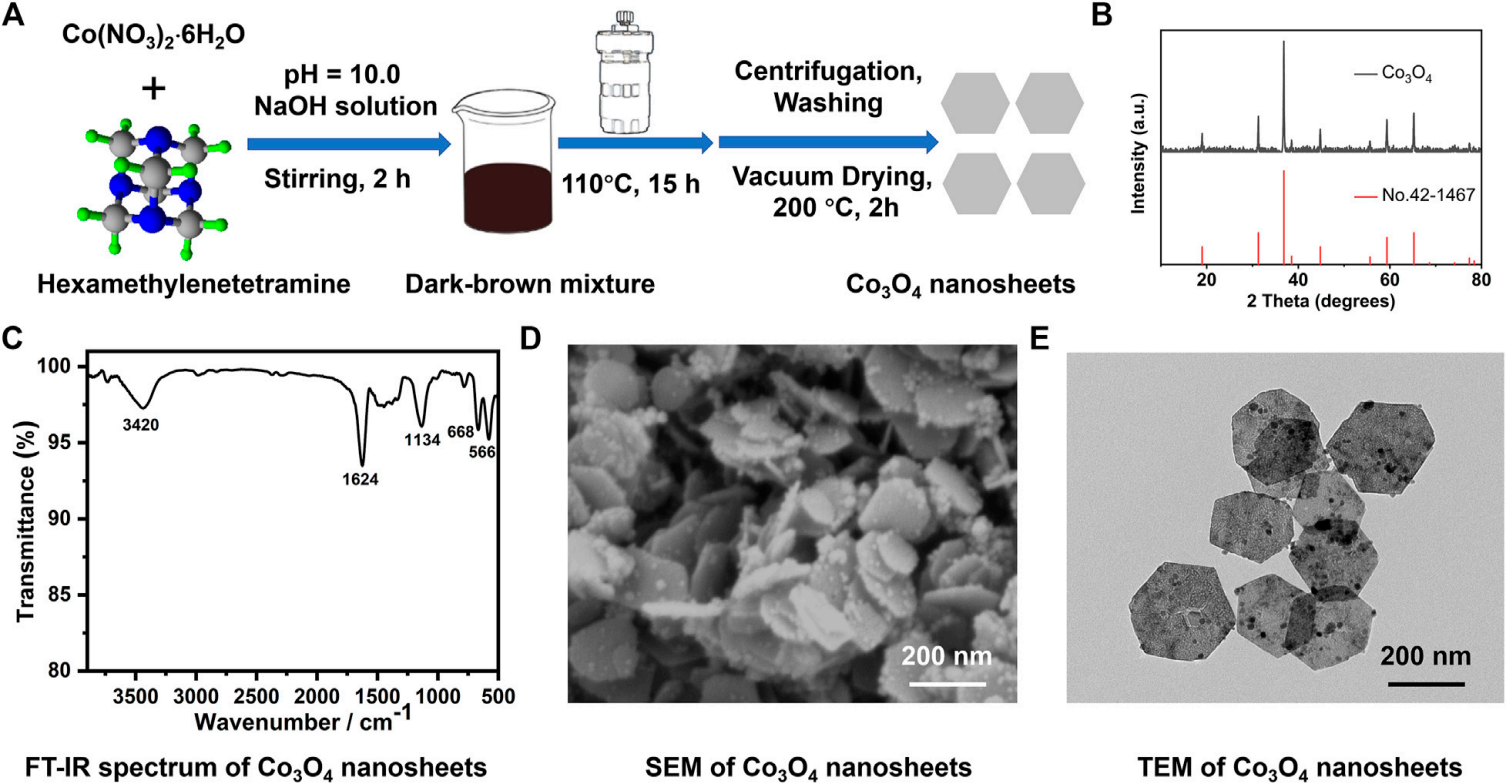
Synthetic process and structural characterizations of the layered Co_3_O_4_ nanosheets **(A)** Illustration for the hydrothermal synthesis of the layered Co_3_O_4_ nanosheets; **(B)** XRD pattern; **(C)** FT-IR spectrum; **(D)** SEM; and **(E)** TEM image of the as-prepared Co_3_O_4_ nanosheets.

Co_3_O_4_ nanosheets were synthesized through a hydrothermal method, using Co(NO_3_)_2_·6H_2_O and hexamethylenetetramine as precursor materials, as shown in Figure 1A. The XRD pattern of the synthetic Co_3_O_4_ nanosheets is shown in Figure 1B. It is obvious that all the characteristic peaks are keeping in with the reported data (JCPDS Card No. 42-1467). The IR spectrum of the Co_3_O_4_ nanosheets is presented in Figure 1C. The two distinct adsorption peaks at 566 cm^−1^ and 668 cm^−1^ are ν1 and ν2, respectively, stretching vibrations of the metal–oxygen bonds which confirm the formation of Co_3_O_4_ (Hwang et al., 2011). The broad absorption bands at 3,420 cm^−1^ and the peak at 1,624 cm^−1^ are due to O-H stretching and the bending vibration of the adsorbed water at the surface. The band at 1,134 cm^−1^ is corresponding to the Co-OH bending vibration. The synthesized nanosheets were also examined by SEM and TEM, which are shown in Figures 1D,E. It is obvious that the synthesized products are hexagonal nanosheets in large quantities. The stacking densities of the nanosheets are very high even though most of them are attached with each other through their surface. Most of them reveal a hexagonal shape with an internal angle of ∼120°; however, there are also some deformed hexagonal-structured nanosheets among them. The nanosheets are about 200 ± 10 nm in diagonal.

### Design Principle of the Biosensor

**FIGURE 2:**
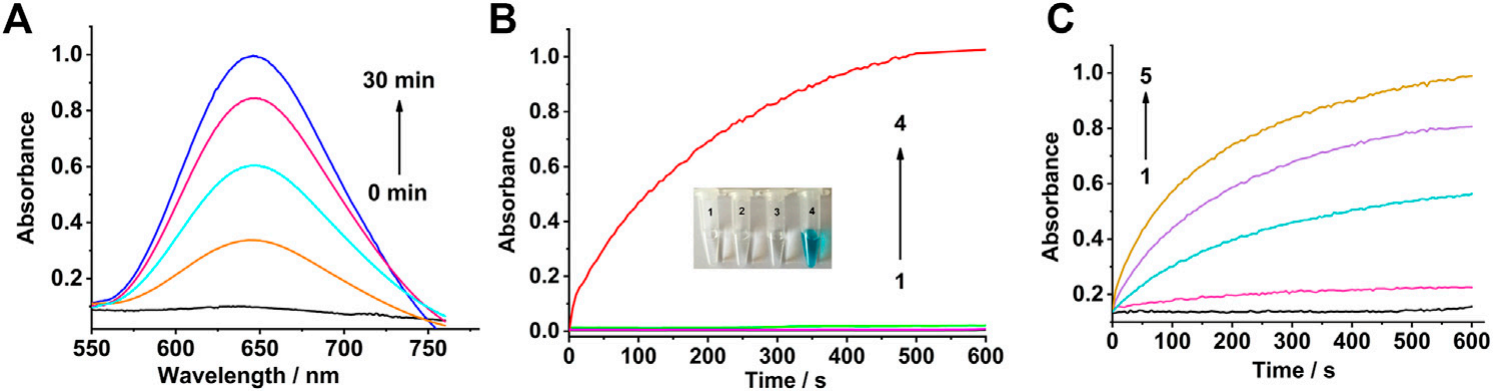
Peroxidase-like activity of the Co_3_O_4_ nanosheets. **(A)** UV–Vis spectra of the mixed solution of TMB, H_2_O_2_, and Co_3_O_4_ nanosheets with different times. **(B)** Time-dependent absorbance changes at 650 nm of TMB solutions in different conditions: (1) Co_3_O_4_ **+** H_2_O_2_, (2) Co_3_O_4_ + TMB, (3) H_2_O_2_ **+** TMB, and (4) Co_3_O_4_ **+** TMB **+** H_2_O_2_. **(C)** Time-dependent absorbance changes at 650 nm in the absence (black) or presence of different concentrations of the Co_3_O_4_ nanosheets: (1)–(5) are 0, 0.5, 5, 10, and 20 μg ml^−1^, respectively.

The colorless TMB solution can be oxidized into a blue solution by H_2_O_2_ in the presence of a catalyst. Therefore, TMB and H_2_O_2_ were used as the reaction substrates to evaluate the peroxidase-like activity of the Co_3_O_4_ nanosheets. The absorbance of oxTMB (the oxidation product of TMB) increased with time in the mixture of Co_3_O_4_ nanosheets, H_2_O_2,_ and TMB (Figure 2A).

It can be seen from Figure 2B that the mixture of Co_3_O_4_ and H_2_O_2_ have no absorption at 650 nm (curve 1). It was also obvious that the oxidation reaction of TMB cannot take place in the presence of Co_3_O_4_ or H_2_O_2_ alone (curves 2 and 3). As a result, TMB was oxidized by H_2_O_2_ in the presence of as-synthesized Co_3_O_4_ nanosheets (curve 4). The absorbance of the mixture at wavelength 650 nm obviously increased with the increasing concentration of the Co_3_O_4_ nanosheets from 0 to 20 μg ml^−1^ which are shown in Figure 2C. As a comparison, the activity of the earlier published peroxidase-like nanomaterials MnO_2_ and 2D Co-MOF (Wang et al., 2022) have also been examined, which are shown in Supplementary Figure S1. To further investigate the catalytic activity of the Co_3_O_4_ nanosheets, the steady-state kinetic parameters were examined for the reaction between H_2_O_2_ and TMB, as shown in Supplementary Figure S2. The results demonstrated that the catalysis of Co_3_O_4_ nanosheets showed typical Michaelis–Menten curves (Supplementary Figure S2A). The curves were fitted to the Lineweaver–Burk plots (Supplementary Figure S2B). According to the plots, the Michaelis–Menten constants (*K*_m_) and the maximum initial reaction rates (*V*_max_) were calculated to be 0.42 mM and 5.7 × 10^−7^ M⋅ s^−1^, respectively, which are superior to the previously published peroxidase-like materials (Lin et al., 2014a; Lin et al., 2014b; Huang et al., 2018). In a word, Co_3_O_4_ nanosheets can facilitate the oxidation of TMB in the presence of H_2_O_2_. So the content of H_2_O_2_ can be detected based on this catalytic reaction principle.

As H_2_O_2_ was the primary catalyzed reaction product of glucose oxidase, the glucose content can also be detected using the Co_3_O_4_ nanosheets as the catalyst (Illustrated as Scheme 1). Additionally, the colorimetric product oxTMB can effectively quench the fluorescence emitted by Ru (bpy)_3_^2+^ due to the IFE. Thus, glucose also can be indirectly detected by the fluorescence method which can not only improve detection sensitivity but also avoid the false positives caused by a single colorimetric signal response (Shin et al., 2020; Munzi et al., 2021; Wan et al., 2021).

### Feasibility Study

**FIGURE 3:**
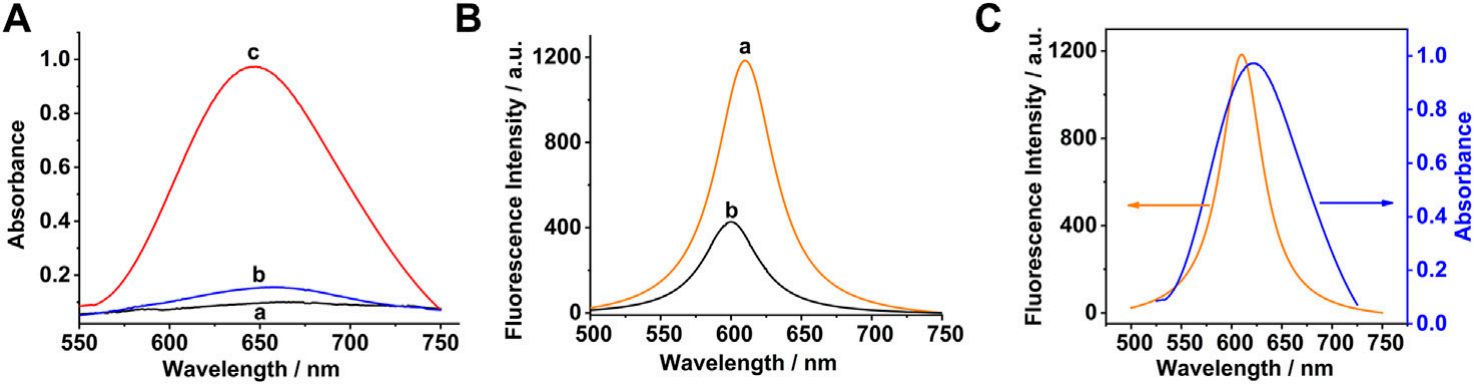
**(A)** Absorbance spectra of TMB under different conditions: **(a)** TMB, **(b)** Co_3_O_4_ + TMB, and **(c)** Co_3_O_4_ **+** TMB **+** H_2_O_2_ **(B)** Fluorescence spectra of Ru (bpy)_3_^2+^ under different experimental conditions: **(a)** Ru (bpy)_3_^2+^; **(b)** Co_3_O_4_ **+** TMB **+** H_2_O_2_ **+** Ru (bpy)_3_^2+^; and **(C)** Fluorescence quenching mechanism. The concentrations of Co_3_O_4_, TMB, H_2_O_2,_ and Ru (bpy)_3_^2+^ were 20 μg/ml, 0.5, 0.05, and 0.5 mM, respectively.

The absorbance spectra of TMB under different conditions were measured to prove the feasibility of the sensing strategy. As shown in Figure 3A, a very weak absorption was obtained in the presence of TMB independently (curve a). There is a slight increase of absorption compared with TMB in the mixture of Co_3_O_4_ and TMB (curve b), which might be a weak catalytic effect of Co_3_O_4_ to TMB. The biggest absorption signal (curve c) was obtained when H_2_O_2_ was added into the aforementioned solution.

Figure 3B shows fluorescence spectra of Ru (bpy)_3_^2+^ under different experimental conditions. As shown in Figure 3B, the biggest fluorescence signal (curve a) was obtained in the presence of Ru(bpy)_3_^2+^ independently. However, the fluorescence intensity significantly decreased when an amount of the Co_3_O_4_ nanosheets**,** TMB, and H_2_O_2_ were added into the aforementioned solution (curve b). It is worth noting that the maximum emission wavelength is blue-shifted slightly. The experimental results demonstrated that a certain amount of oxTMB was produced in the solution, which quenched the fluorescence emitted by Ru(bpy)_3_^2+^ due to the IFE . The fluorescence quenching mechanism of Ru(bpy)_3_^2+^ by the Co_3_O_4_ nanosheets raised from the IFE process are demonstrated in Figure 3C. The reduced fluorescence intensity has a quantitative relationship with the concentration of H_2_O_2_. Thus, the content of glucose can also be detected indirectly by this fluorescence sensing strategy.

### Optimization of Experimental Conditions

To improve the detection sensitivity, the experimental conditions have been optimized. Absorption is dependent on the concentration of the chromogenic reagent, so the concentrations of TMB were initially optimized. The results are shown in Supplementary Figure S3 in SI which showed that the absorption increased gradually with the increase of concentration from 0.1 to 0.4 mM and then increased slightly and kept stable. To ensure the experimental results, therefore, 0.5 mM TMB was selected as the optimal concentration.

All the experimental results confirmed that the Co_3_O_4_ nanosheets revealed a peroxidase-like activity. It was noted that the catalytic activity of peroxidase was associated with the pH and temperature. Therefore, the optimal reaction conditions were investigated. The results demonstrated that Co_3_O_4_ nanosheets have the best catalytic activity in pH 4.5 at 37°C (Supplementary Figures S4, S5 in SI).

The fluorescence detection method in this strategy is based on the inhibition capability of the colorimetric reaction products on the fluorescence emitted by Ru (bpy)_3_^2+^. Therefore, only Ru (bpy)_3_^2+^ concentrations have been optimized to obtain the biggest difference of the fluorescence intensity in the presence of Co_3_O_4_, TMB, H_2_O_2,_ and Ru (bpy)_3_^2+^ compared with Ru (bpy)_3_^2+^ independently. The difference of the fluorescence intensity gradually increased with increasing Ru (bpy)_3_^2+^ concentrations, as shown in Supplementary Figure S6 in SI; the biggest difference was obtained at the concentration of 0.5 mM. Therefore, 0.5 mM Ru (bpy)_3_^2+^ was selected as the optimal concentration for this procedure.

### Colorimetric Detection for H_2_O_2_ and Fluorescence Detection for Glucose

**FIGURE 4:**
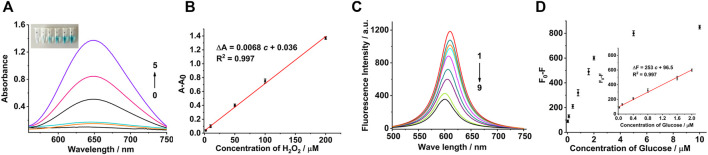
H_2_O_2_ and glucose assays using Co_3_O_4_ nanosheet mimetics. **(A)** UV-Vis absorption spectra in the presence of different H_2_O_2_ concentrations: (0) 0, (1) 2, (2) 10, (3) 50, (4) 100, and (5) 200 μM. The inset photograph shows the visible color change of the reaction system, accordingly. **(B)** Calibration curve corresponding to the absorbance (at the wavelength of 650 nm) as a function of H_2_O_2_ concentration. **(C)** Fluorescence spectra of Ru (bpy)_3_^2+^ in the presence of different concentrations of glucose: (1) 0, (2) 0.02, (3) 0.1, (4) 0.4, (5) 0.8, (6) 1.6, (7) 2.0, (8) 5.0, and (9) 10.0 μM. **(D)** Calibration curve corresponding to the fluorescence intensity (at the emission wavelength of 610 nm) as a function of glucose concentration. Inset shows the linear relationship between the fluorescence intensity and the glucose concentration. The error bars represent the standard deviation of the three measurements. The concentrations of Co_3_O_4_, TMB, and Ru (bpy)_3_^2+^ were 20 μg/ml, 0.5, and 0.5 mM, respectively.

A colorimetric sensor for the detection of H_2_O_2_ was constructed based on the peroxidase-like activity of the Co_3_O_4_ nanosheets. Under the optimal conditions, the concentration-response curve of H_2_O_2_ to TMB at wavelength 650 nm is shown in Figure 4A. A good linear relationship was established between 2 and 200 μM (Figure 4B). The linear regression equation was Δ*A* = 0.0068 *c* + 0.036 with the correlation coefficient of 0.997 [Δ*A*: difference of absorbance and *c*: concentration of H_2_O_2_ (μM)]. The limit of detection for H_2_O_2_ was estimated to be 0.4 μM (based on the signal-to-noise ratio of 3). Similarly, the fluorescence response of the system was examined with different concentrations of glucose which demonstrated that the fluorescence intensity gradually decreased with the increase in the concentration of glucose (Figure 4C). These results verified that with the greater concentration of glucose, more H_2_O_2_ were produced, which inhibited the fluorescence of Ru(bpy)_3_^2+^. The difference of the fluorescence intensity showed a good linear relationship with the concentration of glucose ranging from 0.02 to 2 μM (the inset of Figure 4D). The linear regression equation was Δ*F* = 253 *c* + 96.5 with the correlation coefficient of 0.997 [Δ*F*: difference of fluorescence intensity and *c*: concentration of glucose (μM)]. The limit of detection was estimated to be 5.0 nM (based on the signal-to-noise ratio of 3), which are superior to the reported methods (Supplementary Table S1).

To prove the anti-interference capability of the strategy for the detection of glucose, five other analogs, such as fructose, maltose, sucrose, ascorbic acid, and dopamine, were also determined by the fluorescence method, which are shown in Supplementary Figure S7 in SI. The results showed that the other analogs hardly interfered with the determination of glucose under this experimental condition. The stability of the assay system has also been investigated. Supplementary Figure S8 in SI showed the fluorescence response of the sensing system with the concentration of 0.2 and 1.5 μM glucose based on the six-time detections, respectively. The fluorescence intensity was relatively stable with the relative standard deviation of 2.61 and 3.93%, respectively.

### Determination of H_2_O_2_ and Glucose in Real Samples

H_2_O_2_ in artificial lake water, glucose in fruit juice, and blood samples were detected by colorimetric and fluorescence methods, respectively. The water sample was obtained from the artificial lake in Qingdao Agricultural University, and fruit juice was purchased from the local supermarket. They have been processed according to the literature studies (Wang et al., 2016a; Wang et al., 2016b). Blood samples, provided by the Hospital of Qingdao Agricultural University by collecting from healthy volunteers with informed consent, were processed according to the literature (Peng and Weng, 2017). In addition, the recovery tests were examined by adding a known concentration of the standard to the pretreated solution in real samples (Supplementary Tables S2, S3 in SI). The results demonstrated that the recoveries obtained ranged from about 99.2 to 106.6% and 94–108%, respectively.

## Conclusion

In summary, two-dimensional layered Co_3_O_4_ nanosheets with an intrinsic peroxidase-like catalytic activity have been successfully synthesized by a simple hydrothermal method. The catalytic activity of the Co_3_O_4_ nanosheets has been investigated by the oxidation of TMB by H_2_O_2_ in acidic conditions. The blue oxidation product (oxTMB) was easily visualized and quantified by using a spectrophotometer. Based on this discovery, a simple, cheap colorimetric assay for H_2_O_2_ was successfully developed. Interestingly, we found that the colorimetric product can effectively quench the fluorescence emitted by Ru (bpy)_3_^2+^ due to the IFE. So we further constructed a sensitive and facile fluorescence sensor for the determination of glucose by the catalyzed reaction of glucose oxidase (GOx) with a low detection limit of 5 nM. It has been applied to assay the glucose content in fruit juices and human serum samples. This sensing strategy would facilitate the application of Co_3_O_4_ nanosheets in the fields of biomedicine diagnosis and analytical chemistry.

## Data Availability

The original contributions presented in the study are included in the article/Supplementary Material, further inquiries can be directed to the corresponding author.
