# Supplementary material for: Colorimetric and Fluorescence Dual-Mode Biosensors Based on Peroxidase-Like Activity of the Co3O4 Nanosheets
Source: Front Chem. 2022 Apr 8;10:871013. doi: 10.3389/fchem.2022.871013 (PMC9037028; doi:10.3389/fchem.2022.871013)
Supplement: Supplementary file 1 [file DataSheet1.pdf]

Colorimetric and Fluorescence Dual Modes Biosensor Based on Peroxidase-Like Activity of the Co_3O_4 Nanosheets

Jingying Tan[1#], Weifu Geng[2#], Junde Li[3], Zhen Wang[1], Shaohao Zhu[1], Xiuzhong Wang[1]*

[1]College of Chemistry and Pharmaceutical Sciences, Qingdao Agricultural University, Qingdao, China

[2]College of Plant Health & Medicine, Qingdao Agricultural University, Qingdao, China

[3]Hospital of Qingdao Agricultural University, Qingdao Agricultural University, Qingdao, China

[#] These authors contributed equally to this work.

* Corresponding author: Xiuzhong Wang,

E-mail addresses: xzwang@qau.edu.cn (X. Wang).

Contents

Figure S1. Catalytic activity comparision of Co_3O_4 nanosheets with other materials S3

Figure S2. The steady-state kinetic analysis of Co_3O_4 nanosheets. S4

Figure S3. Optimization experiments of TMB concentration. S5

Figure S4. Optimization experiments of pH S6

Figure S5. Optimization experiments of temperature S7

Figure S6. Optimization experiments of $Ru(bpy)_3^{2+}$ concentration. S8

Figure S7. Selectivity investigation towards different targets S9

Figure S8. The stability of the fabricated fluorescence sensor S10

Table S1. Comparison of various assay methods for glucose S11

Table S2. Colorimetric detection for H_2O_2 in artificial lake water S12

Table S3. Fluorescence detection for glucose in fruit juice and blood samples S13

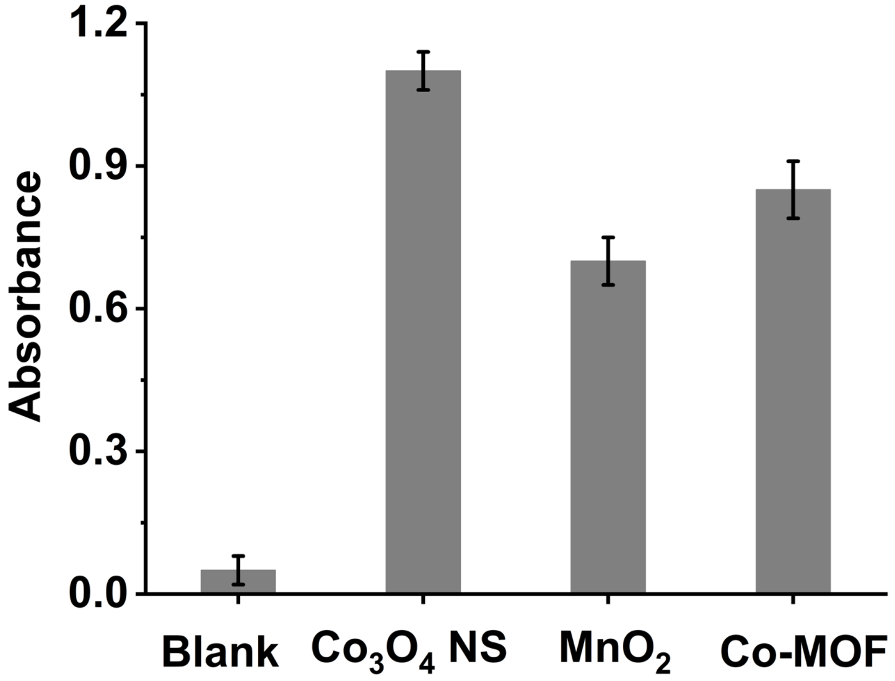

Fig. S1 Catalytic activity of Co_3O_4 nanosheets compared with the earlier published peroxidase-like nanomaterials MnO_2, 2D Co-MOF.

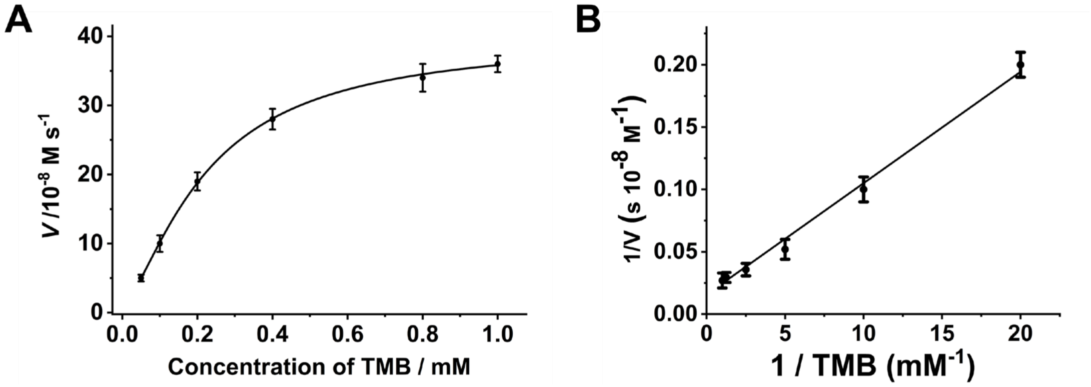

Fig. S2 The steady-state kinetic analysis of Co_3O_4 nanosheets. (A) 5 mM H_2O_2 with different concentration of TMB; (B) Double-reciprocal plots of (A). Experimental conditions: 20 μg·mL^{-1} Co_3O_4 nanosheets in acetate buffer (pH 4.5) at room temperature.

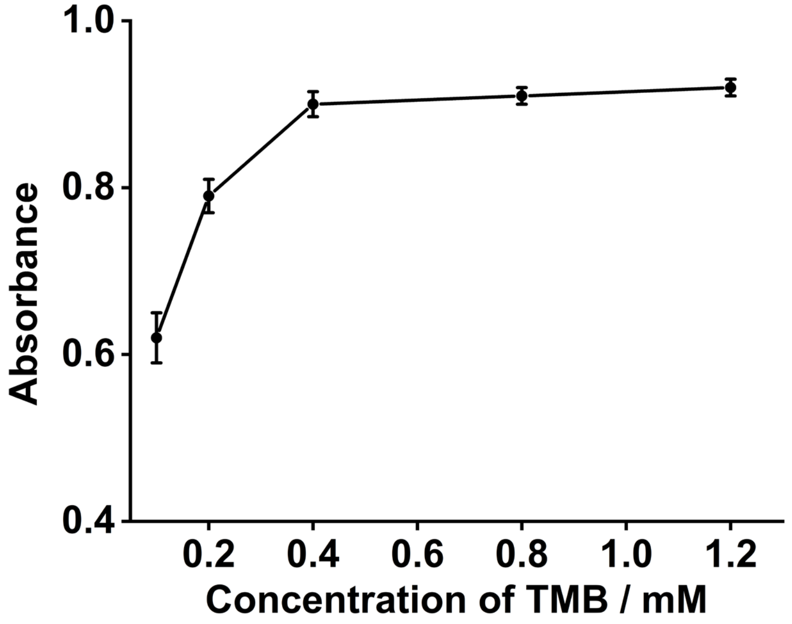

Figure S3. Optimization experiments of TMB concentration. All the colorimetric tests were conducted in HAc-NaAc (0.01 M, pH 4.5) buffer solutions. The concentrations of Co_3O_4 and H_2O_2 were 20 μg/mL and 0.05 mM, respectively.

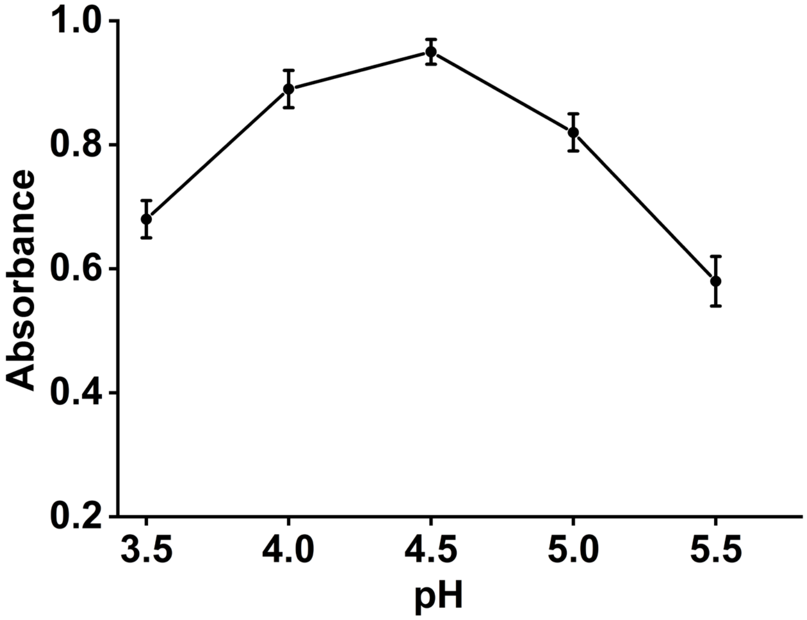

Figure S4. Optimization experiments of pH. All the colorimetric tests were conducted in HAc-NaAc (0.01 M) buffer solutions. The concentrations of Co_3O_4, TMB and H_2O_2 were 20 μg/mL, 0.5 mM and 0.05 mM, respectively.

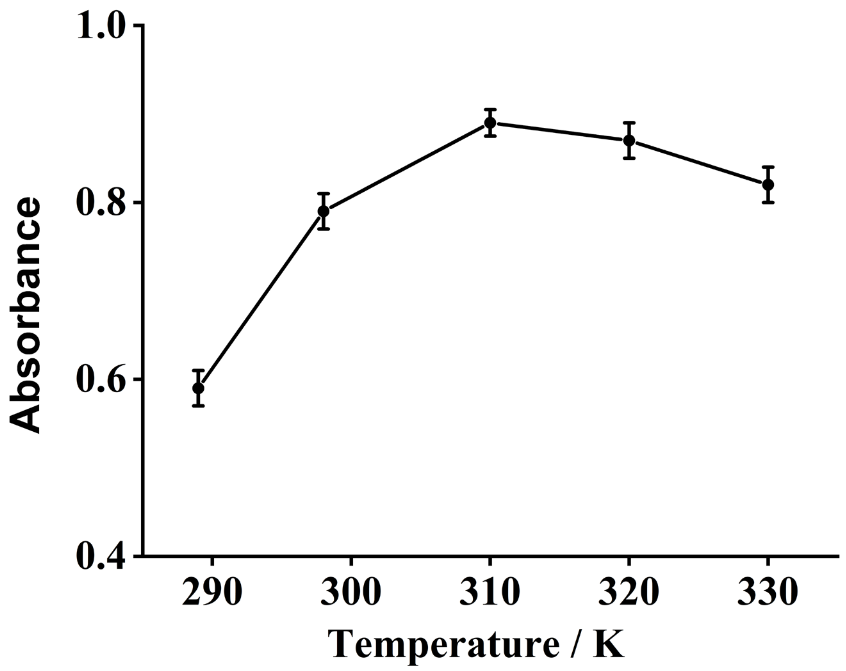

Figure S5. Optimization experiments of temperature. All the colorimetric tests were conducted in HAc-NaAc (0.01 M, pH 4.5) buffer solutions. The concentrations of Co_3O_4, TMB and H_2O_2 were 20 μg/mL, 0.5 mM and 0.05 mM, respectively.

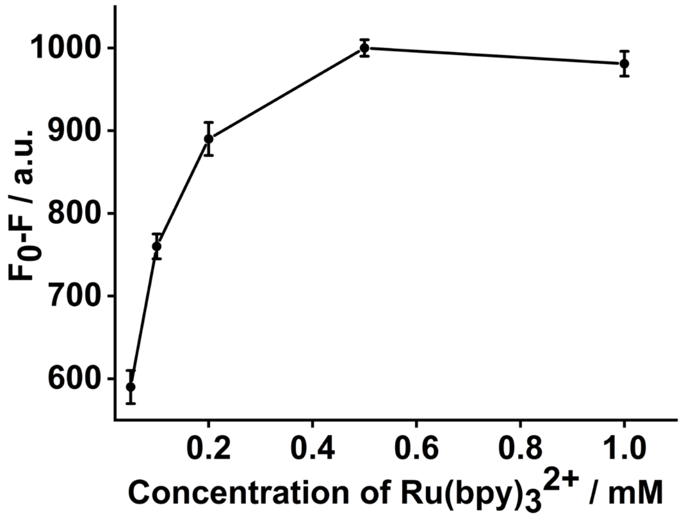

Figure S6. Optimization experiments of $Ru(bpy)_3^{2+}$ concentration. All the colorimetric tests were conducted in HAc-NaAc (0.01 M, pH 4.5) buffer solutions. The concentrations of Co_3O_4, TMB and H_2O_2 were 20 μg/mL, 0.5 mM and 0.05 mM, respectively.

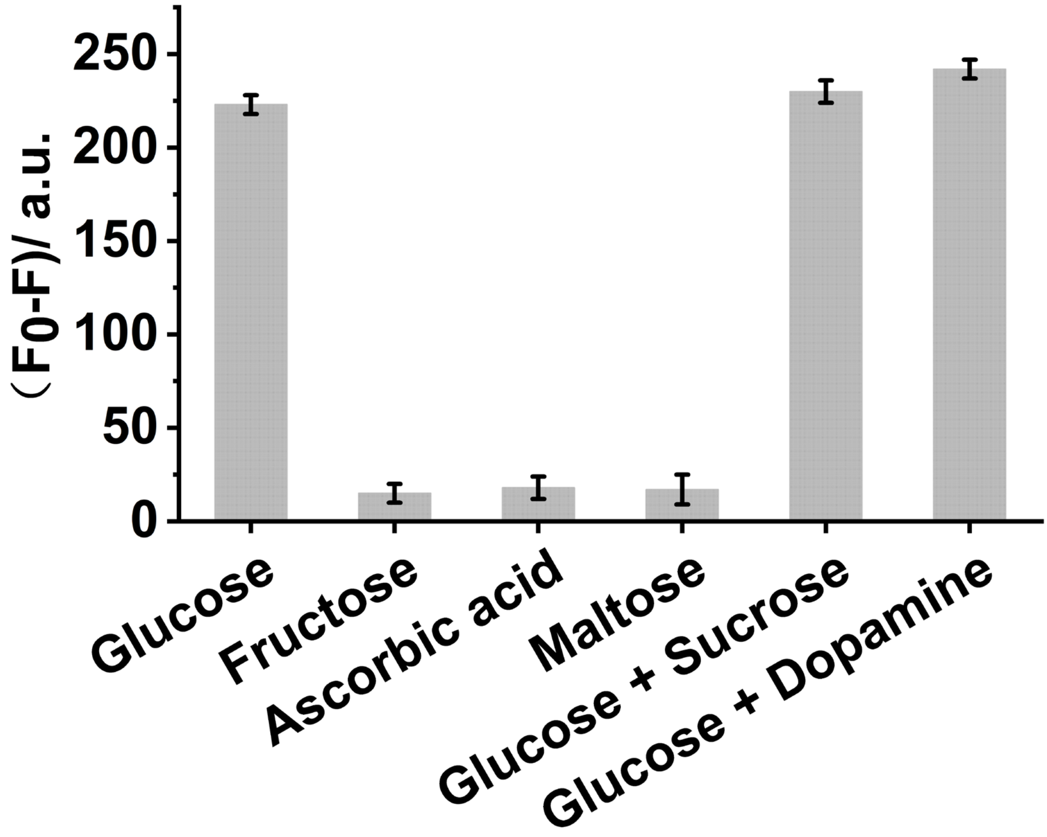

Figure S7. Fluorescence intensity (at the emission wavelength of 610 nm) of the $Ru(bpy)_3^{2+}$ in the presence of glucose, fructose, ascorbic acid, maltose, mixture of glucose and sucrose, mixture of glucose and dopamine, respectively. The concentrations of the above analogue were all 10 μM except that glucose concentration was 0.5 μM. The error bars represent the standard deviation of three measurements.

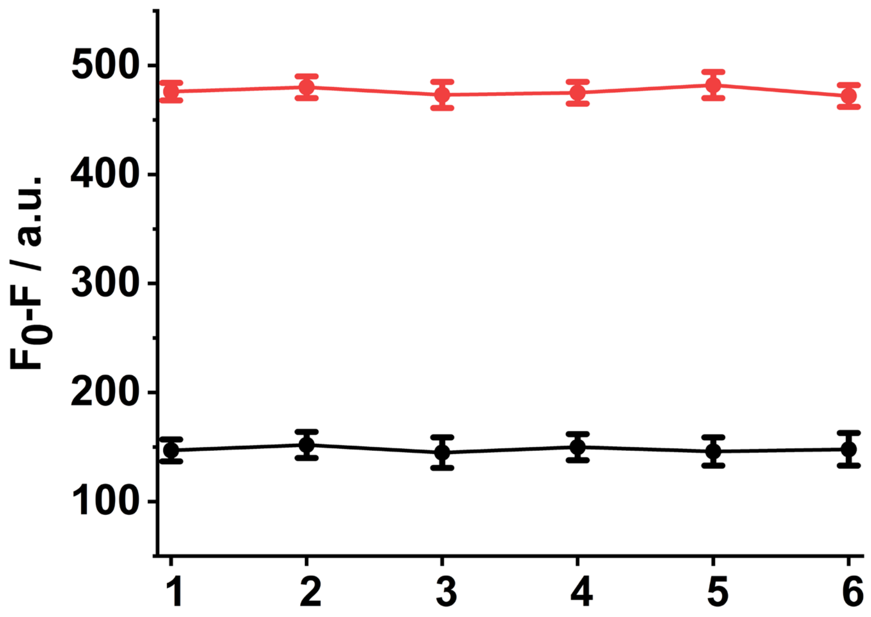

Figure S8. The reproducibility of the fluorescence intensity (at the emission wavelength of 610 nm) of the $Ru(bpy)_3^{2+}$ in the presence of 0.2 and 1.5 μM glucose based on 6 times detections of the proposed biosensing system. The error bars represent the standard deviation of three measurements.

Table S1 Comparison of various glucose assay methods

Detection method	Linear range	LOD	References
Colormetry	5 – 300 μM	2.9 μM	(Lin et al., 2014)
Colorimetry	0.5 – 150 μM	0.2 μM	(Zhang et al., 2021)
Colorimetry	1 – 50 μM	65 nM	(Peng and Weng, 2017)
Electrochemical	0.1– 5.0 mM	0.1μM	(Balouch et al., 2015)
Fluorescence	0.5 – 24 μM	7.54 nM	(Lin et al., 2018)
Fluorescence	0.02 – 2 μM	5.0 nM	This work

References:

Balouch, Q., Ibupoto, Z.H., Khaskheli, G.Q., Soomro, R.A., Sirajuddin, Samoon, M.K., and Deewani, V.K. (2015). Cobalt Oxide Nanoflowers for Electrochemical Determination of Glucose. *J. Electron. Mater.* 44**,** 3724-3732.

Lin, T., Zhong, L., Song, Z., Guo, L., Wu, H., Guo, Q., Chen, Y., Fu, F., and Chen, G. (2014). Visual Detection of Blood Glucose Based on Peroxidase-Like Activity of WS_2 Nanosheets. *Biosens. Bioelectron.* 62**,** 302-307.

Lin, T.R., Qin, Y.M., Huang, Y.L., Yang, R.T., Hou, L., Ye, F.G., and Zhao, S.L. (2018). A Label-Free Fluorescence Assay for Hydrogen Peroxide and Glucose Based on the Bifunctional MIL-53(Fe) Nanozyme. *Chem. Commun.* 54**,** 1762-1765.

Peng, J., and Weng, J. (2017). Enhanced Peroxidase-Like Activity of MoS_2/Graphene Oxide Hybrid with Light Irradiation for Glucose Detection. *Biosens. Bioelectron.* 89**,** 652-658.

Zhang, W.X., Li, X.P., Cui, T.Y., Li, S.C., Qian, Y.Q., Yue, Y., Zhong, W.Y., Xu, B., and Yue, W.Q. (2021). PtS_2 Nanosheets as A Peroxidase-Mimicking Nanozyme for Colorimetric Determination of Hydrogen Peroxide and Glucose. *Mikrochim Acta* 188**,** 174.

Table S2 Colorimetric detection for H_2O_2 in artificial lake water samples

Samples	Amount measured (μM)	Amount added (μM)	Amount measured (μM)	RSD (%) (n=6)	Recovery (%)
Artificial lake water	5.40	5.00	10.73	4.05	106.6
		20.00	25.83	3.71	102.1
		100.00	104.62	6.66	99.2

Table S3 Fluorescence detection for glucose in fruit juice and blood samples

Samples	Amount measured (mM)	Amount added (mM)	Amount measured (mM)	RSD (%) (n=6)	Recovery (%)
Fruit juice 1	335	50.0	389	5.05	108
Fruit juice 2	750	100.0	855	4.12	105
Fruit juice 3	250	50.0	297	4.91	94
Blood sample 1	5.20	5.00	10.55	6.30	107
Blood sample 2	4.90	10.0	14.66	6.68	97.6
